# Supplementary material for: A multiplex blood-based assay targeting DNA methylation in PBMCs enables early detection of breast cancer
Source: Nat Commun. 2023 Aug 7;14:4724. doi: 10.1038/s41467-023-40389-5 (PMC10406825; doi:10.1038/s41467-023-40389-5)
Supplement: Supplementary file 1 — Supplementary Information [file 41467_2023_40389_MOESM1_ESM.pdf]

Supplementary Information

This Supplementary Information File includes:

Supplementary Fig. 1-5

Supplementary Tables 1-9

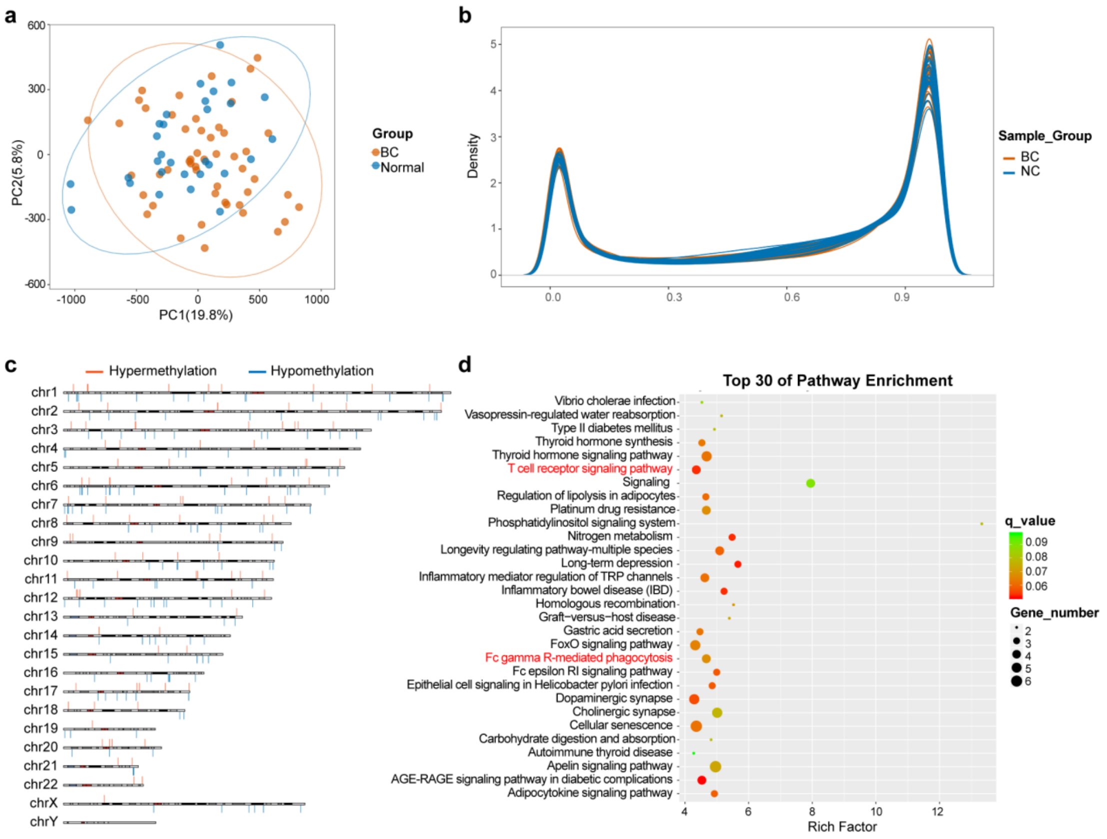

Supplementary Fig. 1 Overview of DNA methylation changes in PBMCs of BC patients compared to the normal controls. a Principal component analysis of whole probes of 850K microarray. Orange, BC; Blue, normal controls. **b** Density plot showing the distribution of the whole array probes for BC and NC samples. **c** The distribution of DMPs on chromosomes. Orange represents hypermethylated DMPs, blue represents hypomethylated DMPs. **d** The KEGG enrichment results of differentially methylated genes showed that multiple pathways were involved in the immune surveillance system. The size of the dot represents the number of differential genes, and the color of the dot represents whether the q value is significant. Source data are provided as a Source Data file.

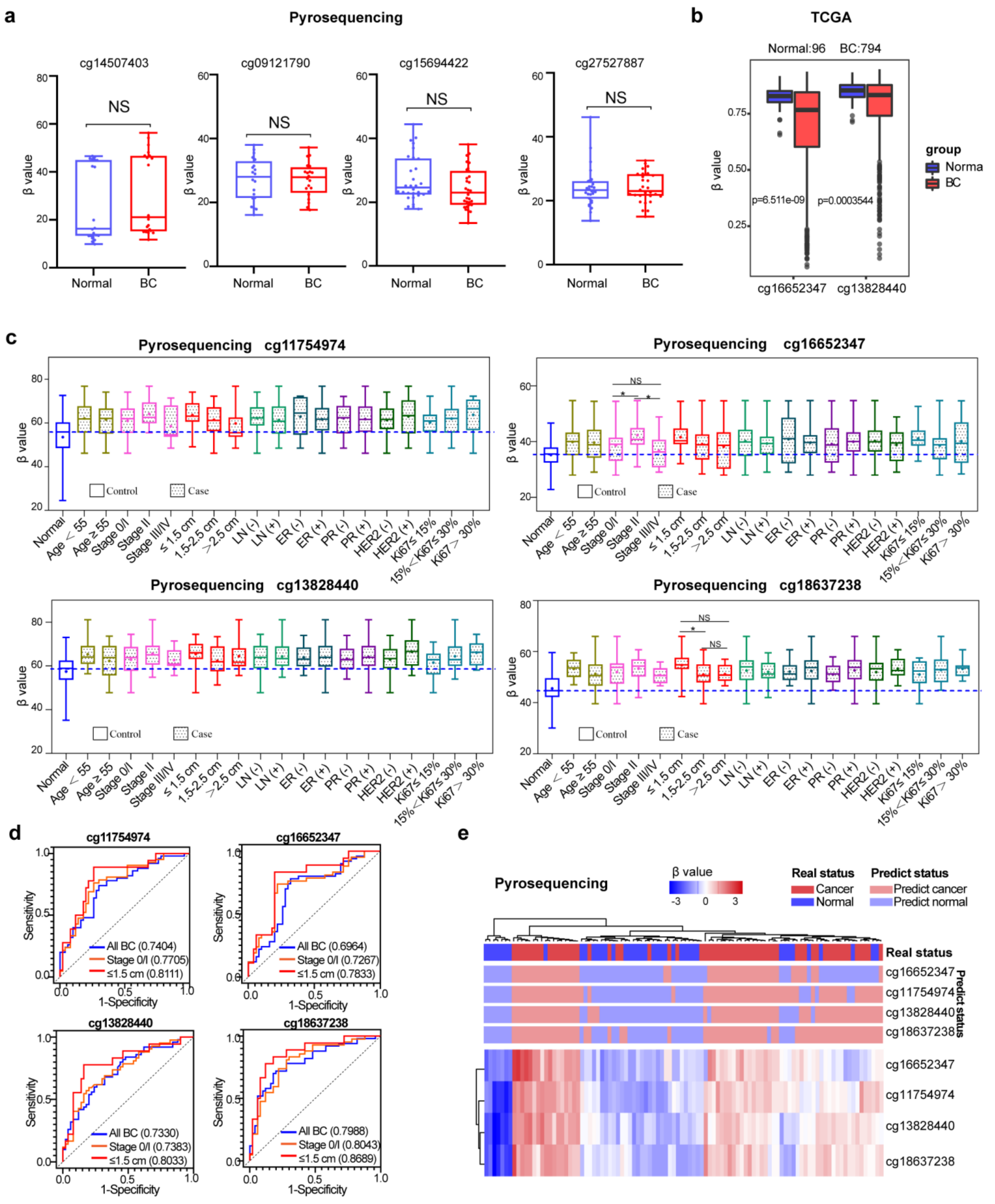

a
Pyrosequencing
cg14507403
cg09121790
cg15694422
cg27527887
NS
β value
Normal
BC
b
TCGA
Normal:96 BC:794
p=6.511e-09
p=0.0003544
group
Norma
BC
cg16652347 cg13828440
c
Pyrosequencing cg11754974
Pyrosequencing cg16652347
Pyrosequencing cg13828440
Pyrosequencing cg18637238
Control
Case
d
cg11754974
All BC (0.7404)
Stage 0/I (0.7705)
≤1.5 cm (0.8111)
cg16652347
All BC (0.6964)
Stage 0/I (0.7267)
≤1.5 cm (0.7833)
cg13828440
All BC (0.7330)
Stage 0/I (0.7383)
≤1.5 cm (0.8033)
cg18637238
All BC (0.7988)
Stage 0/I (0.8043)
≤1.5 cm (0.8689)
Sensitivity
1-Specificity
e
Pyrosequencing
β value
Real status
Cancer
Normal
Predict status
Predict cancer
Predict normal

Supplementary Fig. 2 The validation of BC-associated DNA methylation markers

a The β-value distribution of 4 methylation markers in the pyrosequencing set that were not significantly different between 50 BC patients and 50 normal controls. P values were determined by two-sided Mann-Whitney U test. NS: no significance. **b** The β-value distribution of cg16652347 and cg13828440 in 794 BC and 96 adjacent normal tissue from TCGA database. P values were determined by two-sided Mann-Whitney U test. **c** The methylation levels of 4 methylation markers in 50 normal controls and 50 BC patients of pyrosequencing set with different age, stage, tumor size, lymph node metastasis status, ER status, PR status, HER2 status and Ki-67 levels (One-Way ANOVA test, two-sided, Dunnett's test for multiple comparisons). * $p \leq 0.05$; NS: no significance. **d** ROC curves of 4 methylation markers in all BC, stage 0/I BC and ≤1.5 cm BC of the pyrosequencing set. The AUC for the different categories are shown in the legend. **e** Unsupervised hierarchical clustering of 4 methylation markers based on differential methylation levels between BC patients and normal controls in the pyrosequencing set. The boxes in a, b and c are bounded by the first and third quartile with a horizontal line at the median; minima is the smallest data greater than or equal to the first quartile – 1.5 × interquartile range (IQR); maxima is the largest data point less than or equal quartile + 1.5 × IQR. Source data are provided as a Source Data file.

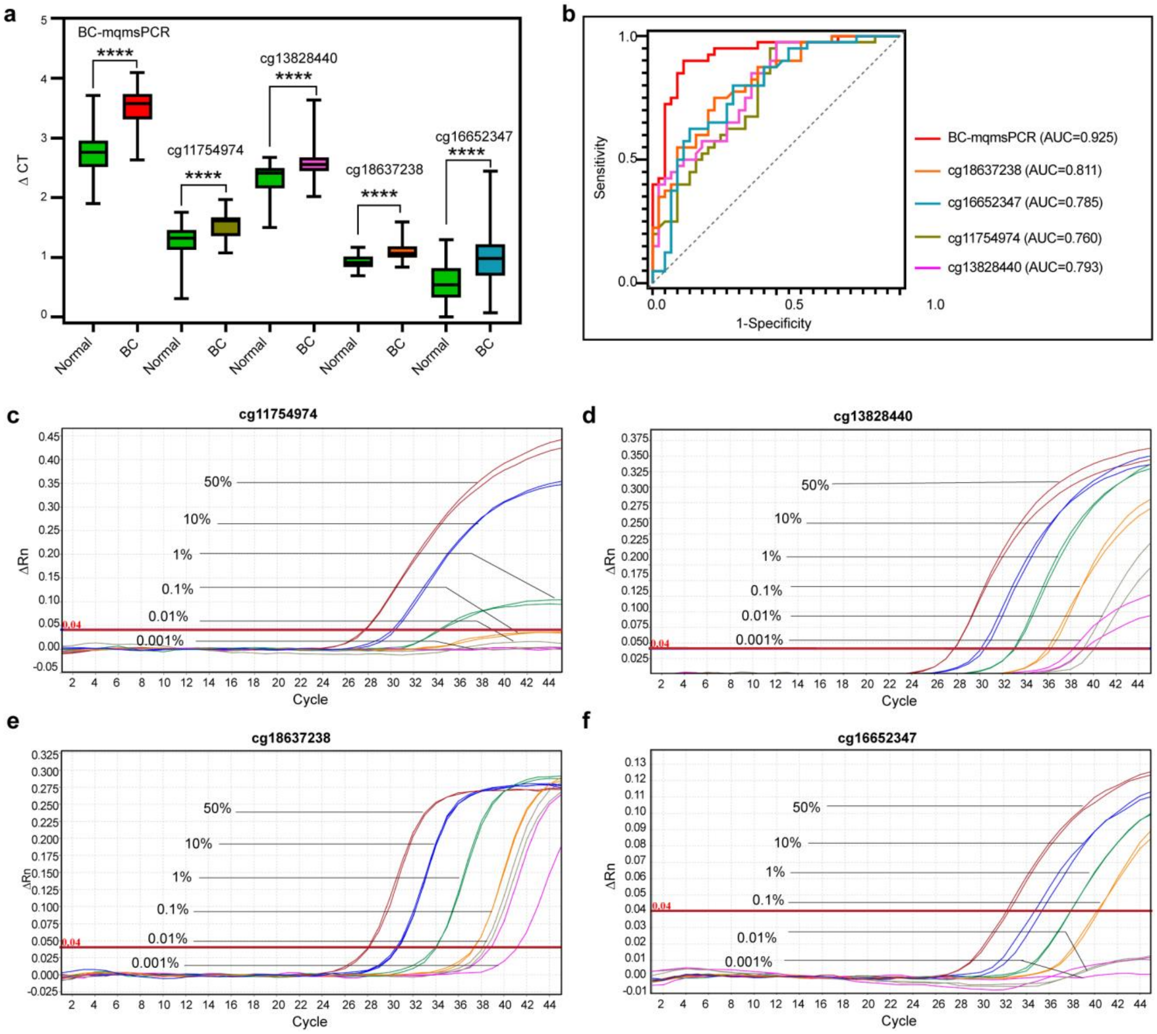

Supplementary Fig. 3 Performance of BC-mqmsPCR compared to individual qMSP assays. **a** The DNA methylation levels of PBMCs were detected by the BC-mqmsPCR assay and cg11754974, cg16652347, cg13828440 and cg18637238 qMSP assay in 40 BC patients and 40 normal controls. The y-axis represents methylation levels ($\Delta CT = CT_{reference} - CT_{biomarker}$), in which a higher value represents a higher methylation level. P values were determined by two-sided Mann-Whitney U test. **** $p \leq 0.0001$. **b** ROC curves of BC-mqmsPCR assay and cg11754974, cg16652347, cg13828440 and cg18637238 qMSP assay for diagnosis of BC. The AUCs for the different categories are shown in the legend. **c** Assessment of the analytical sensitivity of cg11754974 qMSP assay. The assay detected PBMCs DNA signals with as little as 1% of 50ng total PBMCs DNA diluted with pure water. **d** Assessment of the analytical sensitivity of cg13828440 qMSP assay. The assay detected PBMCs DNA signals with as little as 0.1% of 50ng total PBMCs DNA diluted

with pure water. **e** Assessment of the analytical sensitivity of cg18637238 qMSP assay. The assay detected PBMCs DNA signals with as little as 0.1% of 50ng total PBMCs DNA diluted with pure water. **f** Assessment of the analytical sensitivity of cg16652347 qMSP assay. The assay detected PBMCs DNA signals with as little as 0.1% of 50ng total PBMCs DNA diluted with pure water. The boxes in a are bounded by the first and third quartile with a horizontal line at the median; minima is the smallest data greater than or equal to the first quartile – 1.5 × interquartile range (IQR); maxima is the largest data point less than or equal quartile + 1.5 × IQR. Source data are provided as a Source Data file.

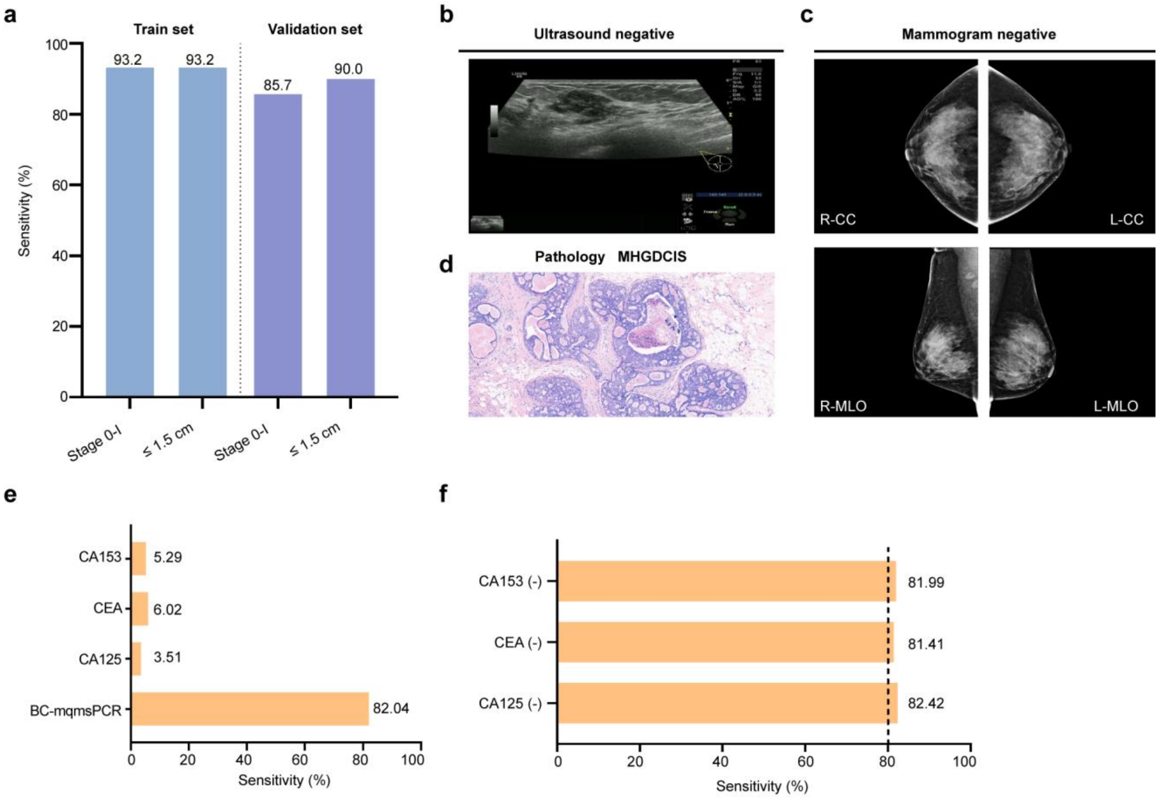

Supplementary Fig. 4 The performance of BC-mqmsPCR in minimal tumor and early-stage BC and the comparison with traditional markers. a In the training and validation sets, the sensitivities of BC-mqmsPCR were 93.2%, 85.7% for stage 0-I BC, and 93.2%, 90.0% for minimal tumor (≤1.5 cm), respectively. **b-d** The ultrasound, mammogram and pathology results of patient 2. The pathology of the tumor was medium to high grade ductal carcinoma in situ (MHGDCIS). The magnifications of the Hematoxylin and eosin (H&E) staining images in pathology were ×200. **e** The performance of BC-mqmsPCR and conventional tumor markers CA153, CEA and CA125. **f** The sensitivity of BC-mqmsPCR in CA125, CEA and CA153 negative breast cancer patients. Source data are provided as a Source Data file.

a

Classification	Predict positive	Predict negative
All	17	153
Lost to follow-up	2	22
In follow-up	15	131
0-1 years after	2	0

b

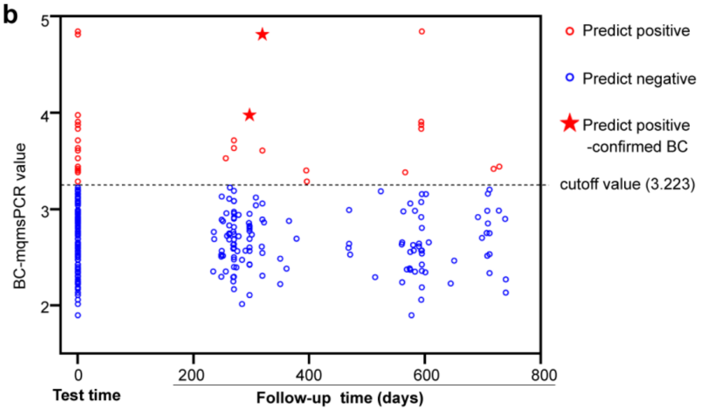

Supplementary Fig. 5 Follow-up results of 170 normal controls. a The follow-up results of 170 normal controls. **b** Scatter plots of follow-up results of 170 normal controls predicted by BC-mqmsPCR. Test time 0 was defined as the BC-mqmsPCR detection time during specimen collection, and follow-up time was defined as the last follow-up time minus specimen collection time. Among the 15 cases with positive prediction, two were diagnosed as breast cancer within 1 year of follow-up; cand among the 131 cases with negative prediction, no confirmed breast cancer was found within 2 years of follow-up. Red hollow circles represent patients with positive prediction who were not diagnosed with breast cancer; blue hollow circles represent patients with negative prediction who were not diagnosed with breast cancer; red solid star represent patients with positive prediction who were diagnosed with breast cancer. Source data are provided as a Source Data file.

Supplementary Table 1. Lasso analysis results

probe	weight
cg25975690	-6.554182
cg21723696	-16.146702
cg15694422	-0.175923
cg14928964	-9.045358
cg02316326	2.366917
cg18881723	-0.194174
cg15354065	-0.167974
cg19774368	-7.443328
cg14770527	-11.624938
cg26582754	-1.001758
cg22997562	-12.213927
cg22748895	-5.871705
cg14507403	-0.270696
cg09821790	-2.18165
cg27527887	-2.925417
cg00301120	-0.285458
cg07420867	3.371767
cg06018119	12.981954
cg22963378	1.849789
cg13972423	-0.621893
cg05158606	-0.131427
cg17513925	-4.907568
cg19142026	4.17347
cg11042561	-0.362002
cg10599438	-2.0518
cg07647771	-0.006002
cg07859923	-11.003146
cg09440129	-0.310301
cg25787588	-2.159916
cg02314201	-0.853294
cg17230281	1.587026
cg02184558	-1.151058
cg02409829	-0.777781

Supplementary Table 2. Differentially methylated CpG positions that meets |Δβ| ≥ 0.08, p value ≤ 0.0001

Probe ID	BC_AVG	NC_AVG	Delta Beta *	P. Value	adjust p value
cg14507403	0.307999642	0.459770748	-0.151771106	4.92E-05	0.037785592
cg14928964	0.388841582	0.502417512	-0.11357593	5.92E-07	0.008336066
cg09821790	0.189663071	0.288357921	-0.09869485	5.12E-05	0.038415334
cg15694422	0.342664856	0.436777967	-0.094113112	2.91E-07	0.007701405
cg27527887	0.323971174	0.410732141	-0.086760967	6.51E-05	0.042723871
cg21723696	0.398814232	0.482918364	-0.084104131	6.23E-08	0.004220147
cg11754974	0.692732536	0.611476452	0.081256084	9.46E-05	0.047762672
cg16652347	0.405147785	0.320770061	0.084377724	2.96E-05	0.030994744

*The Delta Beta is the average β values of the BC group minus the average β values of the normal controls group. The Differential Methylated CpGs Position were calculated by champ.DMP function. The adjust p value were computed using the Benjamini-Hochberg method. Probe ID: identification of the probe (from 850K BeadChip Array); BC: breast cancer; NC: normal controls;

Supplementary Table 3. Summary table of results of two-way ANOVA based on cell components

ID	Pr(>F) Group	F values_Group	levene .F value	levene.p value	shapino.W value	shapino.p value	mean_BC	mean_Normal	sd_BC	sd_Normal
NK cells	0.000685938	12.50715707	1.532939929	0.219385399	0.979227545	0.218495904	0.233244137	0.307490116	0.082656365	0.103360388
Monocytes	0.005408004	8.188995686	3.315194167	0.072477333	0.94984011	0.003375059	0.185993509	0.150786642	0.056042147	0.048236214
Granulocytes	0.205399564	1.630664497	5.365943508	0.023160218	0.955310587	0.007026589	0.104854039	0.083931471	0.078846761	0.055081803
CD4+T cells	0.330760925	0.957826294	0.004804066	0.944919159	0.979370786	0.222993736	0.239582505	0.221635337	0.080228883	0.077995816
B cells	0.477784831	0.508806313	0.192740084	0.661859558	0.974276856	0.106444798	0.088582415	0.083257673	0.033174081	0.030833999
CD8+T cells	0.750400075	0.101912367	0.172116621	0.679376396	0.981844676	0.314824374	0.147743395	0.152898762	0.071118069	0.067867854

P values were determined by two-way ANOVA. BC: breast cancer;

Supplementary Table 4. Table of EWAS results based on Logistic regression models

Probe ID	Estimate	Std Error	Z value	P value
cg11754974	0.176546375	0.07317412	2.412688722	0.015835337
cg16652347	0.225803649	0.067394859	3.350458092	0.00080678
cg13828440	0.261032927	0.114833316	2.273146299	0.023017365
cg18637238	0.238724796	0.10023098	2.381746598	0.01723075
cg14507403	-0.060169757	0.018088697	-3.32637322	0.00087984
cg09821790	-0.061352485	0.030573082	-2.006748455	0.044776454
cg15694422	-0.257659442	0.070673533	-3.645769942	0.000266592
cg27527887	-0.141380207	0.045976006	-3.075086728	0.002104413

P values were determined by t Logistic regression models.

Supplementary Table 5. Confusion tables of binary results of the 4 methylation markers in the targeted bisulfite sequencing and pyrosequencing set

Targeted bisulfite sequencing	cg11754974	cg16652347	cg13828440	cg18637238
Real BC and Predict BC	49	41	50	55
Real BC but Predict Normal	11	19	10	5
Real Normal and Predict Normal	30	37	32	32
Real Normal but Predict BC	10	3	8	8
Sensitivity (%)	81.67	68.33	83.33	91.66
Specificity (%)	75.00	92.50	80.00	80.00
Pyrosequencing	cg11754974	cg16652347	cg13828440	cg18637238
Real BC and Predict BC	35	34	34	40
Real BC but Predict Normal	15	16	16	10
Real Normal and Predict Normal	37	39	34	36
Real Normal but Predict BC	13	11	16	14
Sensitivity (%)	70.00	68.00	68.00	80.00
Specificity (%)	74.00	78.00	68.00	72.00

Supplementary Table 6. Sensitivity of BC-mqmsPCR, CA153, CEA, CA125 to a total of 206 breast cancer patients in the training and validation sets.

Marker	Predict BC	Predict Normal	Sensitivity(%)
CA153	9	161161	5.29
CEA	10	156	6.02
CA125	6	165	3.51
BC-mqmsPCR	169	37	82.04

Supplementary Table 7. Primer sequences used for pyrosequencing

Assay	Primer	5'→3' sequence	5 'modification
cg14507403	cg14507403-15S.F(123bp)	TTTTTGGTTATTTATGTTAGTATTGAGGAG	5'-Biotin
	cg14507403-15S.R	ACACCAAACTCTCCATCAAC	
	cg14507403-15S.S	CAAACACCTATAAATAAATTCC	
cg09821790	cg09821790-16S.F(110bp)	GTATTTATTTGTAGGAGAGGTTGGGTAATG	
	cg09821790-16S.R	ATCTTCCTCTATCTATTCTTCCCTTTTTC	5'-Biotin
	cg09821790-16S.S	GGATAGTTATGTTTTTAGTTAAAAT	
cg15694422	cg15694422-17S.F(96bp)	TTGAGTAATGTATGGGGTATTTTTTAG	
	cg15694422-17S.R	ATAACATCCCCCAAAAAAAAATCCTTACCA	5'-Biotin
	cg15694422-17S.S	GTGGGTAAAAAGAATAGTT	
cg27527887	cg27527887-18S.F(121bp)	GAAGTAATTTTGGTTAGGAAGTTAAGTTG	5'-Biotin
	cg27527887-18S.R	CTACAAACAAACCACTTCTCTTAT	
	cg27527887-18S.S	AACCACTTCTCTTATAAAATAT	
cg11754974	cg11754974-1S.F(228bp)	TGGTGTTGTGTGTTATGGATATAG	5'-Biotin
	cg11754974-1S.R	AACCCTACACAACTATCATTCTCTTTTAC	
	cg11754974-1S.S	TTTACCTAACTCTAACCCAA	
cg16652347	cg16652347-19S.F(66bp)	ATGTGAAGTGTGTATTAAAGGAGTGAA	
	cg16652347-19S.R	ATCCCAATCATTTACAACCTTATCT	5'-Biotin
	cg16652347-19S.S	GTGTATTAAAGGAGTGAAATT	
cg13828440	cg13828440-21S.F(149bp)	ATGAAAATTGAGGTGTTGAAAAAAGTTT	
	cg13828440-21S.R	TTCCATATCTCACTCTATTCTTCTAATCT	5'-Biotin
	cg13828440-21S.S	ATTTATTTAGTTTTATTTTTTTGAA	
cg18637238	cg18637238-22S.F(80bp)	ATGTGGGAGTTTTGTTTTATTAGATAGT	
	cg18637238-22S.R	TATACCCCACATACCACTTTACCTTTA	5'-Biotin
	cg18637238-22S.S	TTTGTTTTATTAGATAGTAAGATTT	

Supplementary Table 8. Primer sequences used for targeted bisulfite sequencing

Assay	Primer	5’→3’ sequence
cg11754974	cg11754974_F	TGTGTGTTATGGATATAGGTTAGAGAAGTT
	cg11754974_R	TAATAACCCTACACAACTATCATTCTCTTT
cg13828440	cg13828440_F	GAATGAGGYGTTTAAGTGGGAAA
	cg13828440_R	TTATACTTAACACAATTTAAAACACTTAACATATC
cg16652347	cg16652347_F	GGATGTTAAGAGTAAGGGTGTTTG
	cg16652347_R	ATTAACACTTTCCTCACCTCCTTT
cg18637238	cg18637238_F	GTTAAGATTAGGAAAGTTATAATTGAAGATG
	cg18637238_R	TTTCCCACTTTATCTACAATATTACCTT

Supplementary Table 9. Primer and probe sequences for the BC-mqmsPCR assay

Assay	primer and probe	5'→3' sequence
cg18637238	cg18637238_F	GTTAAGATTAGGAAAGTTATAATTGAAGATG
	cg18637238_R	CACATACCACTTTACCTTTATTAATACGA
	cg18637238_Probe	ACATAAATCTTACTATCTAATAAAACAAAACTCCCACA
cg13828440	cg13828440_F	AATAAGGAAAATAAAGAAGTTGAAGTCG
	cg13828440_R	TACCTAATTATCCTAACCACGACAAAC
	cg13828440_Probe	AATAAAACTAAATAAATTTCCCACTTAAACGCC
cg16652347	cg16652347_F	TTTTAAAGTTTATTATAGGAAGAGGTTGTT
	cg16652347_R	CAATCATTTACAACCTTATCTTTAACG
	cg16652347_Probe	CACTCCTTTAATACACACGTCACATAAAACG
cg11754974	cg11754974_F	TTTTTTGTTTGGTTTTAGTTTAACG
	cg11754974_R	ATACAATATAACATAAAAACCATATAAATTACTC
	cg11754974_Probe	CTCATCACCAAAAATCAAAATCGTTAAAC
ACTB	ACTB_F	TGGTGATGGAGGAGGTTTAGTAAGT
	ACTB_R	AACCAATAAAACCTACTCCTCCCTTAAA
	ACTB_Probe	ACCACCACCCAACACACAATAACAAACACA
