# Supplementary material for: A multiplex blood-based assay targeting DNA methylation in PBMCs enables early detection of breast cancer
Source: Nat Commun. 2023 Aug 7;14:4724. doi: 10.1038/s41467-023-40389-5 (PMC10406825; doi:10.1038/s41467-023-40389-5)
Supplement: Supplementary file 3 — Description of Additional Supplementary Files [file 41467_2023_40389_MOESM3_ESM.pdf]

Description of Additional Supplementary Files

Supplementary Data 1
Description:Demographic and clinical characteristics of study participants

Supplementary Data 2
Description:Demographic and clinical characteristics of training and validation cohort

Supplementary Data 3
Description:Follow-up results of 170 healthy controls
