# Supplementary material for: A multiplex blood-based assay targeting DNA methylation in PBMCs enables early detection of breast cancer
Source: Nat Commun. 2023 Aug 7;14:4724. doi: 10.1038/s41467-023-40389-5 (PMC10406825; doi:10.1038/s41467-023-40389-5)
Supplement: Supplementary file 7 — Reporting Summary [file 41467_2023_40389_MOESM7_ESM.pdf]

nature portfolio

Corresponding author(s): Chuanxin Wang, Lutao Du, Juan Li

Last updated by author(s): Jul 13, 2023

Reporting Summary

Nature Portfolio wishes to improve the reproducibility of the work that we publish. This form provides structure for consistency and transparency in reporting. For further information on Nature Portfolio policies, see our Editorial Policies and the Editorial Policy Checklist.

Statistics

For all statistical analyses, confirm that the following items are present in the figure legend, table legend, main text, or Methods section.

n/a	Confirmed	
☐	☒	The exact sample size (n) for each experimental group/condition, given as a discrete number and unit of measurement
☒	☐	A statement on whether measurements were taken from distinct samples or whether the same sample was measured repeatedly
☐	☒	The statistical test(s) used AND whether they are one- or two-sided<br>*Only common tests should be described solely by name; describe more complex techniques in the Methods section.*
☐	☒	A description of all covariates tested
☐	☒	A description of any assumptions or corrections, such as tests of normality and adjustment for multiple comparisons
☐	☒	A full description of the statistical parameters including central tendency (e.g. means) or other basic estimates (e.g. regression coefficient) AND variation (e.g. standard deviation) or associated estimates of uncertainty (e.g. confidence intervals)
☒	☐	For null hypothesis testing, the test statistic (e.g. F, t, r) with confidence intervals, effect sizes, degrees of freedom and P value noted<br>*Give P values as exact values whenever suitable.*
☒	☐	For Bayesian analysis, information on the choice of priors and Markov chain Monte Carlo settings
☒	☐	For hierarchical and complex designs, identification of the appropriate level for tests and full reporting of outcomes
☒	☐	Estimates of effect sizes (e.g. Cohen's d, Pearson's r), indicating how they were calculated

Our web collection on statistics for biologists contains articles on many of the points above.

Software and code

Policy information about availability of computer code

Data collection	The Infinium Human Methylation 850K BeadChip analysis was performed according to the manufacturer's instructions, and the data were analyzed using the ChAMP package in R (4.0.0).
Data analysis	R 4.0.0, ChAMP 2.18.2, ggplot2 3.3.0, SVA 3.36.0, PyroMark Assay Design Software 2.0, Pyro Q-CpG Software

For manuscripts utilizing custom algorithms or software that are central to the research but not yet described in published literature, software must be made available to editors and reviewers. We strongly encourage code deposition in a community repository (e.g. GitHub). See the Nature Portfolio guidelines for submitting code & software for further information.

Data

Policy information about availability of data

All manuscripts must include a data availability statement. This statement should provide the following information, where applicable:

- Accession codes, unique identifiers, or web links for publicly available datasets
- A description of any restrictions on data availability
- For clinical datasets or third party data, please ensure that the statement adheres to our policy

Genome-wide DNA methylation data from the discovery phase are deposited in the Gene Expression Omnibus (GEO accession No: GSE237036). The original data presented in graphs generated in this study are provided in the Supplementary Information and Source Data file. Source data are provided with this paper.

Human research participants

Policy information about studies involving human research participants and Sex and Gender in Research.

Reporting on sex and gender	All study participants were female.
Population characteristics	The cases were selected by being (i) Pathological diagnosis of BC (without any treatment such as surgery and radiotherapy and chemotherapy); (ii) No other cancers were present; (iii) Subject are no other known inflammatory diseases (bacterial or viral infections, asthma, autoimmune diseases, active thyroid disease) that may alter PBMCs characteristics; (iv)Subjects can understand and sign written informed consent to participate in the study. Controls were free of malignant diseases and were frequency matched to cases on age and race.
Recruitment	A total of 820 patients were enrolled in this study from 10 hospitals in 6 provinces of China between May 2020 and July 2022. The cases were selected by being (i) Pathological diagnosis of BC (without any treatment such as surgery and radiotherapy and chemotherapy); (ii) No other cancers were present; (iii) Subject are no other known inflammatory diseases (bacterial or viral infections, asthma, autoimmune diseases, active thyroid disease) that may alter PBMCs characteristics; (iv)Subjects can understand and sign written informed consent to participate in the study. Controls were free of malignant diseases and were frequency matched to cases on age and race. All methylation tests were conducted in PBMCs samples. Because blood samples were collected prior to pathological diagnosis and clinical treatments, 39 samples were excluded from analysis due to: 1. the lack of pathological data; 2. pathologically confirmed benign lesions; 3. insufficient amount of DNA extracted from PBMCs; 4. low quality tests. The remaining 781 PBMCs samples (366 BC, 290 normal controls and 125 other tumors) were used for DNA methylation profiling, methylation marker screening, and development of novel methods for early BC diagnosis.
Ethics oversight	The corresponding author, on behalf of all authors, jointly and severally, certifies that their institution has approved the protocol for any investigation involving humans and that all experimentation was conducted in conformity with ethical and humane principles of research.

Note that full information on the approval of the study protocol must also be provided in the manuscript.

Field-specific reporting

Please select the one below that is the best fit for your research. If you are not sure, read the appropriate sections before making your selection.

- [x] Life sciences
- [ ] Behavioural & social sciences
- [ ] Ecological, evolutionary & environmental sciences

For a reference copy of the document with all sections, see nature.com/documents/nr-reporting-summary-flat.pdf

Life sciences study design

All studies must disclose on these points even when the disclosure is negative.

Sample size	In the discovery phase, we included 50 breast cancer patients and 30 healthy controls for genome-wide methylation analysis. In the multistep validation phase, we included 110 breast cancer cases and 90 healthy controls for DNA methylation marker validation. At the stage of assay development and application, 501 samples (206 BC, 170 normal controls and 125 other tumors) were included for methodological establishment and verification, without sample size calculation.
Data exclusions	39 samples were excluded from analysis due to: 1. the lack of pathological data; 2. pathologically confirmed benign lesions; 3. insufficient amount of DNA extracted from PBMCs; 4. low quality tests.
Replication	No replication due to limited amount of clinical samples.
Randomization	There were no randomization in the study design.
Blinding	Not applicable as this was not a randomized clinical trial.

Reporting for specific materials, systems and methods

We require information from authors about some types of materials, experimental systems and methods used in many studies. Here, indicate whether each material, system or method listed is relevant to your study. If you are not sure if a list item applies to your research, read the appropriate section before selecting a response.

Materials & experimental systems

n/a	Involved in the study	
[x]	[ ]	Antibodies
[x]	[ ]	Eukaryotic cell lines
[x]	[ ]	Palaeontology and archaeology
[x]	[ ]	Animals and other organisms
[ ]	[x]	Clinical data
[x]	[ ]	Dual use research of concern

Methods

n/a	Involved in the study	
[x]	[ ]	ChIP-seq
[x]	[ ]	Flow cytometry
[x]	[ ]	MRI-based neuroimaging

Clinical data

Policy information about clinical studies

All manuscripts should comply with the ICMJEguidelines for publication of clinical research and a completedCONSORT checklist must be included with all submissions.

Clinical trial registration	This project has been registered in the Chinese Clinical Trial Registry.
Study protocol	Detailed protocols are provided in Materials and Methods.
Data collection	See Materials and Methods.
Outcomes	The pathological diagnosis of breast cancer was used as the gold standard.
